# The Comparative Analysis of Genomic Diversity and Genes Involved in Carbohydrate Metabolism of Eighty-Eight *Bifidobacterium pseudocatenulatum* Isolates from Different Niches of China

**DOI:** 10.3390/nu14112347

**Published:** 2022-06-04

**Authors:** Guopeng Lin, Qian Liu, Luyao Wang, Haitao Li, Jianxin Zhao, Hao Zhang, Gang Wang, Wei Chen

**Affiliations:** 1 State Key Laboratory of Food Science and Technology, Jiangnan University, Wuxi 214122, China; 7200112079@stu.jiangnan.edu.cn (G.L.); 6170112047@stu.jiangnan.edu.cn (Q.L.); 6190112161@stu.jiangnan.edu.cn (L.W.); liht@jiangnan.edu.cn (H.L.); zhaojianxin@jiangnan.edu.cn (J.Z.); zhanghao61@jiangnan.edu.cn (H.Z.); chenwei66@jiangnan.edu.cn (W.C.); 2 School of Food Science and Technology, Jiangnan University, Wuxi 214122, China; 3 Institute of Food Biotechnology, Jiangnan University, Yangzhou 225004, China; 4 National Engineering Research Center for Functional Food, Jiangnan University, Wuxi 214122, China; 5 Wuxi Translational Medicine Research Center and Jiangsu Translational Medicine Research Institute, Wuxi Branch, Wuxi 214122, China

**Keywords:** *Bifidobacterium pseudocatenulatum*, genomic diversity, phylogenetic relationship, comparative genomics, carbohydrate metabolism

## Abstract

Eighty-eight *Bifidobacterium pseudocatenulatum* strains, which were isolated from human, chicken and cow fecal samples from different niches of China, were compared genomically in this study to evaluate their diversity. It was found that *B. pseudocatenulatum* displayed a closed pan-genome, including abundant glycoside hydrolase families of the carbohydrate active enzyme (CAZy). A total of 30 kinds of glycoside hydrolases (GHs), 14 kinds of glycosyl transferases (GTs), 13 kinds of carbohydrate-binding modules (CBMs), 6 kinds of carbohydrate-esterases (CEs), and 2 kinds of auxiliary activities (AAs) gene families were identified across the genomes of the 88 *B. pseudocatenulatum* strains. Specifically, this showed that significant differences were also present in the number of 10 carbohydrate-active enzyme gene families (GT51, GH13_32, GH26, GH42, GH121, GH3, AA3, CBM46, CE2, and CE6) among the strains derived from the hosts of different age groups, particularly between strains from infants and those from other human age groups. Twelve different individuals of *B. pseudocatenulatum* from four main clusters were selected for further study to reveal the genetic diversity of carbohydrate metabolism-related genes within the same phylogenetics. The animal experiment showed that 3 weeks of oral administration and 1 week after cessation of administration of these strains did not markedly alter the serum routine inflammatory indicators in mice. Furthermore, the administration of these strains did not significantly cause adverse changes in the gut microbiota, as indicated by the α- and β-diversity indexes, relative to the control group (normal diet). Beyond that, FAHBZ9L5 significantly increased the abundance of *B. pseudocatenulatum* after 3 weeks and significantly increased the abundance of acetic acid and butyric acid in the host’s intestinal tract 3 and 4 weeks after the first administration, respectively, compared with the control group. Corresponding to this, comparative genomic analyses of 12 *B. pseudocatenulatum* suggest that FAHBZ9L5-specific genes were rich in ABC transporters and carbohydrate esterase. Combining the results of comparative genomics analyses and animal experiment, it is suggested that the strains containing certain gene clusters contribute to another competitive growth advantage of *B. pseudocatenulatum,* which facilitates its intestinal carbohydrate metabolism in a host.

## 1. Introduction

*Bifidobacterium pseudocatenulatum* can be found in the intestinal tract of people of all ages, but particularly in that of nursing infants [1,2]. Compared with other *Bifidobacterium* species, *B. pseudocatenulatum* has been more strongly associated with metabolic diseases in both animal and human studies, which have demonstrated its importance in modulating immune functions, lipid metabolism, and glycolipid metabolism [3,4,5]. Studies have demonstrated that the administration of the strain *B. pseudocatenulatum* CECT7765 or *B. pseudocatenulatum* CAG0046 could ameliorate metabolic and immunologic obesity-associated alterations [6,7]. In addition, some strains of *B. pseudocatenulatum* produce beneficial compounds, such as urolithin and conjugated linoleic acid [8,9,10], indicating that they are potentially probiotic strains. Although many strains of *B. pseudocatenulatum* have probiotic functions, there has been little research on the genetic diversity and evolutionary relationships of this species.

Few studies have investigated the genome of *B. pseudocatenulatum* and even fewer have included multiple strains, so the genomic diversity of this species remains largely unexplored. A comparative genomic analysis of four strains of *B. pseudocatenulatum* and six strains of *B. catenulatum* revealed that the genomic differences between the two species were mainly reflected in niche-related genes, such as CRISPR loci, fimbrial genes, and exopolysaccharide-related genes. In addition, there were 1355 shared genes between the 2 species, which were mainly related to carbohydrate metabolism and material transport [11]. Corresponding to the prior findings, the ability to use complex carbohydrates facilitates the stable colonization of the *Bifidobacterium* species in the human gut [12]. Another study showed that the average nucleotide identity (ANI) values of strains from the same habitat were as high as 99.88%, while those of strains from different habitats were as low as 97.80% [3]. Thus, even distantly related species may show a high ANI of >96%. It can be observed that the phylogenetic relationship of *B. pseudocatenulatum* can be accurately understood only by the whole-genome alignment of this species. However, the relationship between the niche and genomic diversity of *B. pseudocatenulatum* strains remains poorly understood to date.

In this study, we analyzed their pan-genome and core-genome, as well as their phylogenetic tree based on single copy orthologous genes of 88 *B. pseudocatenulatum* strains. The raw data of 88 *B. pseudocatenulatum* strains contain the previous comparative genomic information of 66 *B. pseudocatenulatum* strains [13]. Since we did not have enough previous research as the basis for estimating sample size, we chose 66 genomes of *B. pseudocatenulatum* strains investigated in our previous study and 22 additional genomes of *B. pseudocatenulatum* strains in this work to assess the adequacy of the analysis for this experiment. Furthermore, we tried to explore the relationships between the abundance of special genes involved in carbohydrate metabolism and difference placement strains within the phylogenetic tree, to better understand the evolutionary relationships for *B. pseudocatenulatum*. In summary, the present study investigated the genomic diversity of *B. pseudocatenulatum* by comparative genomic analysis, combined with its impact on the gut microbiota, to speculate the possible contribution of specific carbohydrate metabolism genes to some of the strain’s probiotic functions, with a view to provide recommendations for the screening and application of probiotic strains.

## 2. Materials and Methods

### 2.1. Probiotic Strains and Culture

Fecal samples were collected from 397 healthy volunteers and animals. The selection of population samples covered different regions and ages in China. Participants or their legal guardians provided written informed consent for the use of their fecal samples. Isolation and screening of 22 *B. pseudocatenulatum* were anaerobically performed with modified MRS (De Man, Rogosa and Sharpe) medium (containing 0.05% L-cysteine hydro-chloride and 100 mg/L of mupirocin) at 37 °C. This genome was sequenced using the Illumina HiSeq 1000 pair-end approach by Shanghai Major Biotechnology Co., Ltd. (Shanghai, China) and the final assembly resulted in a draft genome (Table S1). The draft genomes of the other 66 *B. pseudocatenulatum* strains were displayed in our previous study [13]. The amino acid sequences of 120 *B. pseudocatenulatum* strains were downloaded from the NCBI Assembly Database. Then, the amino acid sequences related to carbohydrate metabolism were selected from the genomes to construct a database. The amino acid sequences of 12 distinct individuals of *B. pseudocatenulatum* from the 4 main clusters of the phylogenetic tree were selected for BLAST with the previously constructed database (sequence identity > 70%, match length > 40%, https://github.com/linguopeng/article/tree/master/Fig_4c (26 April 2022)). A total of 12 strains of *B. pseudocatenulatum* were cultured for 3 successive generations in MRS liquid medium, each inoculation amount was 4% (*v*/*v*), anaerobic culture at 37 °C for 24 h, 500 mL of the bacterial liquid was taken, centrifuged at 8000× *g* for 5 min, and finally, the bacteria were collected for animal experiments.

### 2.2. Genome Assembly, Genome Annotation and Pangenome Analysis

Reads were assembled by SOAPdenovo and local inner gaps were filled by using the software GapCloser (https://github.com/BGI-Qingdao/TGS-GapCloser/ (v1.0.1, BGI, Qingdao, China) (26 April 2022)) [14]. The calculations of the reads’ content of genomes files were performed using SeqKit tools (https://bioinf.shenwei.me/seqkit/ (v2.1.0) (26 April 2022)). Genome assemblies were evaluated using QUAST (quality assessment tool for genome assemblies, QUAST v4.6.3). Overall, for each strain, Prodigal (v2.6.3), Glimmer (v3.02) and GeneMarkS-2 (web-server) were run on their default settings to generate sets of predicted genes [15,16]. The predicted genes were annotated in public databases, including the non-redundant (NR, download on 31 July 2020), Kyoto Encyclopaedia of Genes and Genomes (KEGG, http://www.genome.jp/kegg (release 58) (26 April 2022)), Carbohydrate-Active Enzyme database (CAZy, http://www.cazy.org (release 5.0) (26 April 2022)). Additionally, the eggNOG-mapper (https://github.com/eggnogdb/eggnog-mapper (v2.1.6) (26 April 2022)) was also used for further annotation. In brief, the Pan-Genome analysis pipeline (https://hub.docker.com/repository/docker/linguopeng/pgap (v1.12) (26 April 2022)), which adapts the dockerfile from https://github.com/kastman/pgap-docker/ (v1.12) (26 April 2022)), was used to analyze the pan-genome. For all the genomes used in this study, pan-genome calculation was performed using PGAP (v1.12). in order to calculate the total gene repertoire encountered in the newly sequenced *B. pseudocatenulatum,* and the degree of overlap and diversity, with respect to other publicly available genomes. An average nucleotide identity (ANI) analysis was performed using FastANI (v1.33) [17]. All the protein sequences were compared using all-against-all alignments. Then, the orthologous genes were clustered using OrthoMCL (v2.0.9) and OrthoFinder (https://github.com/davidemms/OrthoFinder/ (v2.5.4) (26 April 2022)) [18]. The genes that were common among all the 88 *B. pseudocatenulatum* strains were defined as orthologous genes that presented at least once in each genome assayed. In addition, the genes consisting of those presenting in some but not each genome made up the variable or dispensable genes. All of the orthologous genes were categorized in predicted functional groups based on COG (Clusters of Orthologous Groups, http://www.ncbi.nlm.nih.gov/COG (version 2003) (26 April 2022)). The orthologous genes of all the *B. pseudocatenulatum* genomes were aligned using MAFFT v7.3. Phylogeny inference package v3.6 was used to construct the phylogenetic tree, based on the single copy genes. The final phylogenetic tree with their associated data was visualized using the ggtree R package (v1.14.6) [19].

### 2.3. Animals and Experimental Design

C57BL/6 mice (6 weeks of age, purchased from SLAC experimental animal Co., Ltd., Shanghai, China) were raised in independent cages with alternating administration under 12-h light/12-h dark cycle at 24 ± 1 °C. The animal experiment was in accordance with the regulations of the experimental animal ethics committee of Jiangnan university (qualified number: JN. No. 20191115 c0840115[319]). After one week of the adaptation period, the mice were gavaged the concentration of surviving bacteria at 10^9^ CFU/mL at a dose of 0.1 mL/10 g body weight. In this study, 12 distinct individuals of *B. pseudocatenulatum* from the 4 main clusters of the phylogenetic tree were used for animal treatment. Detailed experimental design was shown in the Supplementary Materials Table S2.

### 2.4. Biochemical Parameters

High-density cholesterol (HDL-C), low-density lipoprotein cholesterol (LDL-C), total cholesterol (TC) and triacylglycerols (TG) levels were measured with commercial assay kits purchased from Jiancheng Bioengineering Institute (Nanjing, China). The serum lipid biochemical indices were measured using an automatic biochemical analyzer (Hitachi, Japan). Inflammatory cytokines tumor necrosis factor-α (TNF-α), interleukin 10 (IL-10), interleukin 1 beta (IL-1β), interleukin 6 (IL-6) and interleukin 12 (IL-12) in serum were determined using ELISA kits (Bangyi, Shanghai, China), following the manufacturer’s protocols.

### 2.5. Gut Microbiota Sequencing and Short Chain Fatty Acids (SCFA) Determination

Briefly, the mice fecal genomic DNA was extracted using the FastDNA SPIN Kit for Soil (MP Biomedicals, CA, USA). Each genomic DNA sample was divided into two parts. For one part, the V3–V4 hypervariable regions of 16S rRNA gene were amplified using specific primers (forward primer 341F: 5′-CCTAYGGGRBGCASCAG-3′, reverse primer 806R: 5′-GGACTACNNGGGTATCTAAT-3′). For another part, as described previously [20], the extracted fecal genome was used as a template to specifically amplify the *GroEL* gene fragment of all the species within the *Bifdobacterium* genus using the following primers: Bif-*GroEL*-F (5′-TCCGATTACGAYCGYGAGAAGCT-3′) and Bif-*GroEL*-R (5′-CSGCYTCGGTSGTCAGGAACAG-3′) to further determine the abundances of all the species belonging to the *Bifidobacterium* genus in feces. The final library was sequenced by paired-end MiSeq (2 × 300 cycle configuration). The raw Illumina paired-end reads data were determined by QIIME2 (https://qiime2.org/ (v2019.7) (26 April 2022)). The pre-trained 16S rRNA classifier (silva-132-99-nb-341F-806R_classifier.qza, https://hub.docker.com/repository/docker/linguopeng/qiime2_bif_lac (v2) (26 April 2022)) and *Bifdobacterium* classifier (Bif_F_R_classifier.qza, https://github.com/linguopeng/article/blob/master/Bif_F_R_classifier.qza (26 April 2022)) were used to perform the taxonomic classification. Alpha and beta diversity measures were calculated using the Qiime pipeline (https://github.com/linguopeng/article/blob/master/Qiime2-2019.7pipeline.sh (26 April 2022). A complete image of a Linux OS with all the dependencies and the pipeline scripts is available from dockerhub (https://hub.docker.com/repository/docker/linguopeng/qiime2_bif_lac (v2) (26 April 2022)) for more detail. The concentrations of SCFAs in the fecal samples were measured by GC-MS analysis, according to the method by Guangsu Zhu et al. (2018) [21].

### 2.6. Statistical Analysis

The data are presented as mean ± standard deviation (SD). All statistical analyses were performed in R (version 4.0.3). The comparisons between groups were performed by one-way analysis of variance (ANOVA). The ‘multcomp’ package was used to perform post hoc multiple comparison testing using Tukey’s HSD. Completely different lowercase letters indicate significant differences at *p* < 0.05.

## 3. Results

### 3.1. General Features of the Genomes of B. pseudocatenulatum Strains

The general features of the genomes of 88 *B. pseudocatenulatum* strains are presented in Table S1. The genome sizes ranged from 1.98 to 2.75 Mbp, with an average of 2.24 ± 0.10 Mbp, which was close to that of this species previously published in the NCBI database (2.2 Mbp, RefSeq: GCF_020541885.1) and the average genome size of *B. adolescentis* (2.23 Mbp) [22]. The number of predicted tRNA genes of *B. pseudocatenulatum* strains was an average of 55 ± 3. The number of predicted open reading frames (ORFs) ranged from 1811 to 2674, with an average of 1950. The ANI values of whole genomes between homologous regions shared by any two genomes of these strains mentioned above are generally greater than 96% (Figure S1).

### 3.2. B. pseudocatenulatum Genomic Diversity

**Figure 1:**
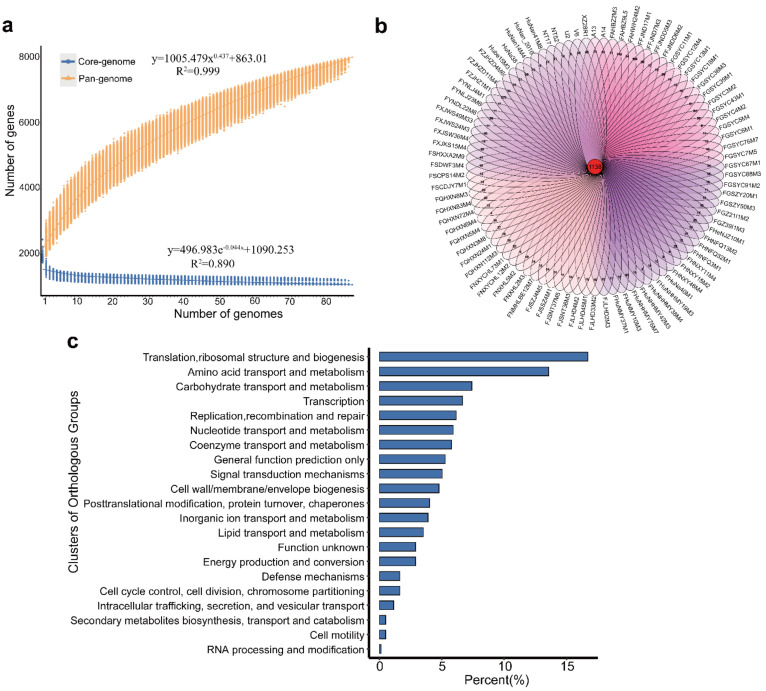
Pan−genome and core−genome of the 88 strains of *B. pseudocatenulatum* (**a**), Venn diagram representing core genes and unique genes of 88 *B. pseudocatenulatum* strains (**b**), Clusters of Orthologous Groups (COG) annotation of the core genes within 88 *B. pseudocatenulatum* (**c**).

Gene accumulation curves for the pan-genome (orange) and core-genome (blue) (Figure 1a) show the diversity and stability of the genomes across the strains. The number of pan-genome genes from the 88 *B. pseudocatenulatum* strains increased logarithmically with the number of genomes analyzed. We obtained 7977 genes from the genomes of 88 *B. pseudocatenulatum* strains, which was approximately 4.57 times the number of genes in a single *B. pseudocatenulatum* strain (GCF_020541885.1). According to the HEAP rule [23], the curve index of the pan-genome was 0.437, which suggested that the 88 *B. pseudocatenulatum* strains showed an open but soon to be closed pan-genome. The number of core genes in *B. pseudocatenulatum* remained stable with the increasing number of strain genomes, and no conspicuous changes occurred when the number of strain genomes increased from 20 to 30. (Figure 1a). The number of core genes of 88 *B. pseudocatenulatum* strains was 1138 (Figure 1b), accounting for about 60% of the total genes in every strain, while the proportion of non-essential genes in each strain was as high as 40%. Among the 88 *B. pseudocatenulatum* strains, those with the most unique genes were FZJHZ1M1 screened from 7-day-old males, FaHBZ9L5 from 3-year-old males, and FFJND17M1 from 3-month-old males. These strains exhibited 273, 143, and 124 strain-specific genes, respectively.

Core genes of the 88 *B. pseudocatenulatum* strains were extracted for COG annotation analysis (Figure 1c). Most of these genes were involved in fundamental mechanisms of cell functioning that are crucial for survival. The results of the core gene function analysis were similar to those of previous studies on *Bifidobacterium* [24]. The number of shared genes involved in the metabolism of important substances (e.g., amino acids, carbohydrates, and nucleotides) was the largest, accounting for approximately 40% of the core genome. Among them, carbohydrate transport- and metabolism-related genes comprised 7.4% of the core genome, which corresponds to the prior findings that the ability to use complex carbohydrates facilitates the stable colonization of *Bifidobacterium* species in the human gut [12]. In addition, some core genes with unknown functions were also found.

### 3.3. Evolutionary Genomic Diversity, the GH Enzymes, Orthogroups Unassigned and Antibiotic Resistance Genes of B. pseudocatenulatum

**Figure 2:**
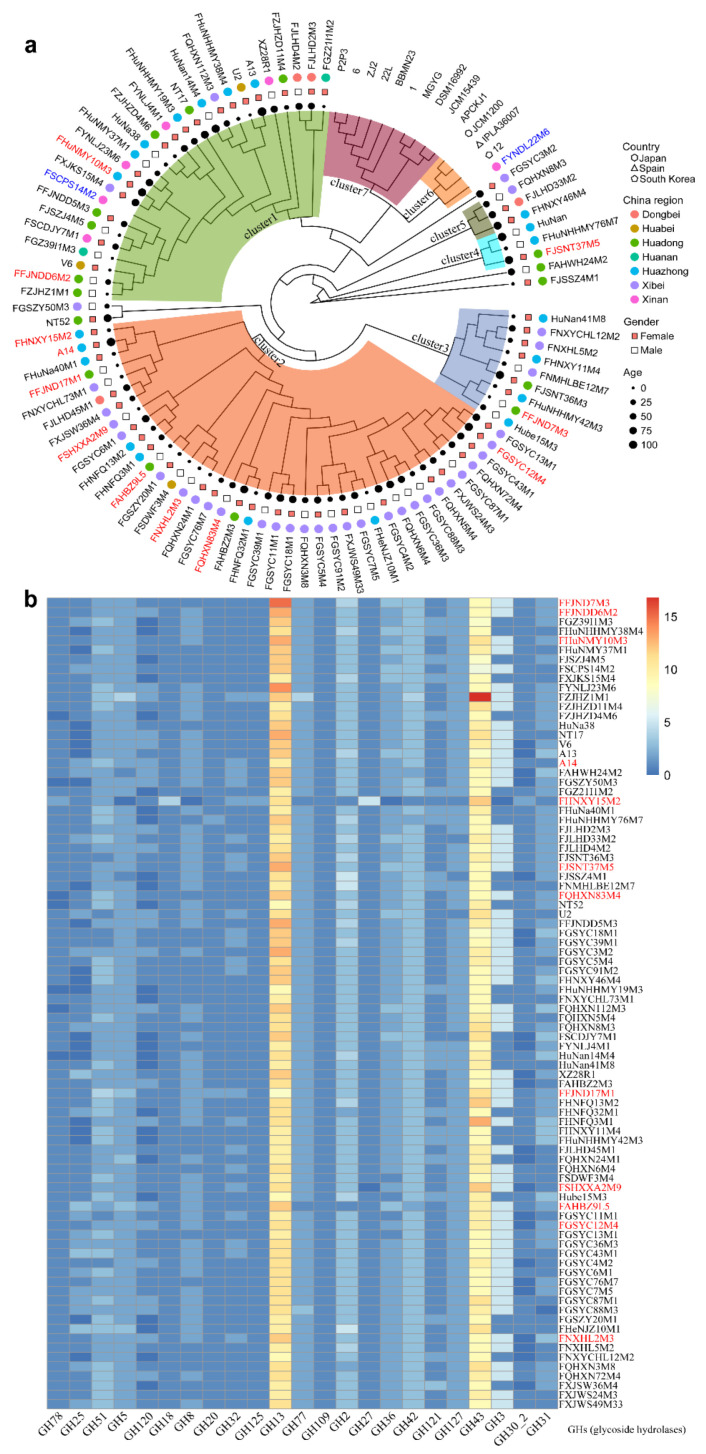
Phylogenetic tree based on single copy gene orthologous group and distribution of carbohydrate-active enzyme within 88 *B. pseudocatenulatum* strains (**a**,**b**). The color scales represent the number of GH (glycoside hydrolases) families that existed in each strain, with blue representing the absence of matches and red squares representing the highest number of GH families.

*B. pseudocatenulatum*, *B. adolescentis*, and *B. catenulatum* are closely related bacterial species of the genus *Bifidobacterium*, which is dominant in the intestinal microbiota of adult humans. To explore the evolutionary relationships among these three *Bifidobacterium* species and within the strains of *B. pseudocatenulatum*, we constructed a phylogenetic tree based on the genes of the 88 *B. pseudocatenulatum* strains we isolated, as well as the orthologous genes of 3 *B. pseudocatenulatum* strains, 4 *B. catenulatum* strains, and 6 *B. adolescentis* strains published in the NCBI database (Supplementary Material Table S3). A total of 765 single copy gene orthologous groups were obtained (https://github.com/linguopeng/article/blob/master/Single_Copy_Orthologue_Sequences.zip (26 April 2022)). In addition, the sequences of single copy genes from each species were concatenated into a super gene orthologous to infer the phylogeny tree. *B. adolescentis* had a distant genetic relationship with *B. pseudocatenulatum*, while *B. catenulatum* had a close genetic relationship with *B. pseudocatenulatum*. Among the *B. pseudocatenulatum* strains, FYNDL22M6 isolated from cow feces was the most closely related to the other strains published in the NCBI database. FSCPS14M2 isolated from chicken feces was also clustered with the human FFJNDD5M3 strain, suggesting that the omnivorous diet of chickens and these relationships may suggest that ecological variables, such as habitat and feeding mode, are important variables that may influence the subsequent evolution of *B. pseudocatenulatum* in organisms (Figure 2a). The most of reference strains were all derived from fecal samples of non-Chinese group populations; therefore, the NCBI reference *B. pseudocatenulatum* strains were distant from the other Chinese clusters. The results indicated that the 88 *B. pseudocatenulatum* strains isolated from various niches of China exhibited their unique phylogenetic patterns.

Considering its potential relationship with the colonization ability of *B. pseudocatenulatum* in the gut, to investigate the utilization of various carbon sources by *B. pseudocatenulatum* strains, we annotated and analyzed the 88 strains based on the CAZy database and created a heatmap of carbohydrate-active enzyme gene families (Figure 2b) to visually display their distribution across the studied strains. A total of 30 kinds of glycoside hydrolase (GHs), 14 kinds of glycosyl transferase (GTs), 13 kinds of carbohydrate-binding module (CBMs), 6 kinds of carbohydrate-esterase (CEs), and 2 kinds of auxiliary activity (AAs) gene families were identified across the genomes. In accordance with its position of cluster6 in the phylogenetic tree, the animal-derived strain FYNDL22M6 lacked the GH18, GH125, GH121, and GH127 gene families, which are related to the use of arabinose-derived substrates and close to the strains derived from the fecal samples of non-Chinese group populations.

**Figure 3:**
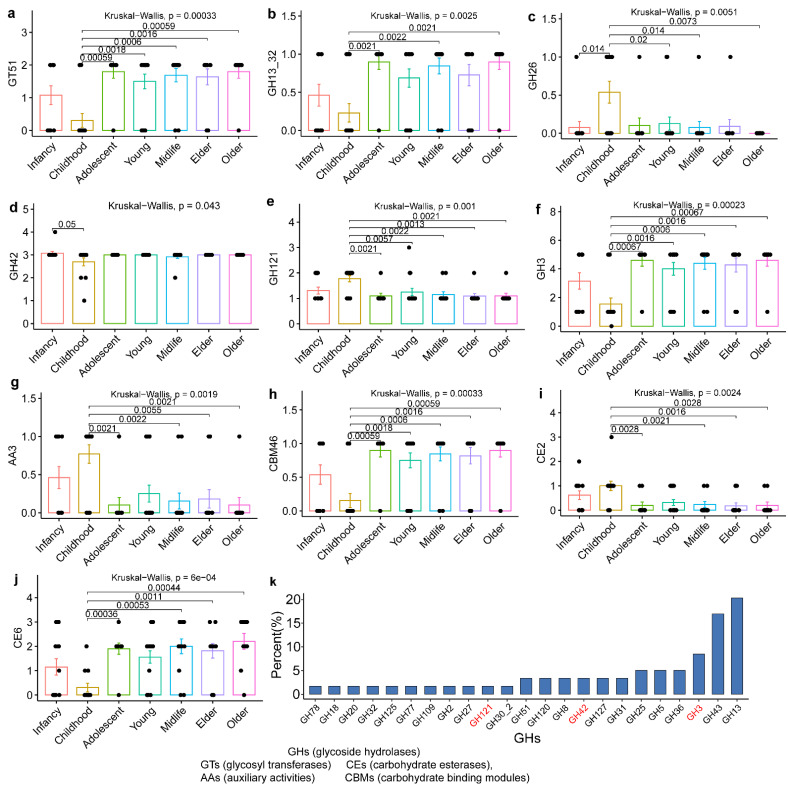
The number of carbohydrate-active enzyme in different age groups (**a**–**j**: infancy: 0–1.5 years, childhood: 1.5–3.5 years, adolescent: 4–18 years, young: 21–45 years, midlife: 47–60 years, elder: 65–79 years, older: 80–108 years; GHs (glycoside hydrolases), GTs (glycosyl transferases), CEs (carbohydrate esterases), AAs (auxiliary activities) and CBMs (carbohydrate binding modules)). Percent (%) as percentage of all GHs within 88 *Bifidobacterium pseudocatenulatum* strains. Each solid black dot represents a strain. (**k**). Significant testing was performed using Wilcoxon rank sum test (*p* < 0.05). Red words as a significant difference marker of GHs in different age groups.

**Figure 4:**
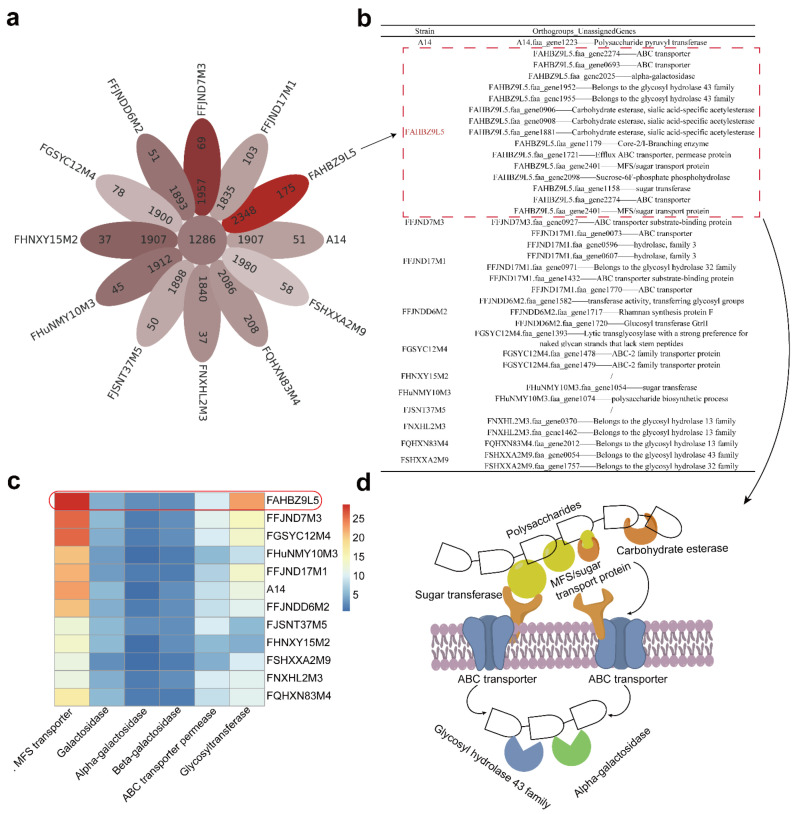
(**a**) The flower plot displays the core orthogroups’ number (in the center), the sum number of genes of species, and the number of orthogroups’ unassigned genes (in the petals) for the 12 *B. pseudocatenulatum*. (**b**) A set of related carbohydrate metabolism genes (orthogroups unassigned genes) of 12 *B. pseudocatenulatum*. (**c**) Heatmap representation accounts for gene numbers of related carbohydrate metabolism. (**d**) FAHBZ9L5-specific genes of *B. pseudocatenulatum* encoding proteins predicted to be involved with polysaccharide utilization.

To further explore the differences in carbohydrate utilization among the strains across different host age groups, we used the Kruskal–Wallis test to analyze the differences in the number of genes encoding active enzymes for different carbohydrates across the samples from different host age groups. This showed that the following 10 gene families were specific to host age: GT51, GH13_32, GH26, GH42, GH121, GH3, AA3, CBM46, CE2, and CE6 (Figure 3). Differences in the number of genes encoding carbohydrate-active enzymes across the age groups were mainly found between the strains derived from infants and strains from other age groups. Notably, significant differences were fewest between the lactation host and the infant host, which were limited to the abundance of GH26 and GH42 gene families. The GH gene families occupied a large proportion of the carbohydrate-active enzyme gene families across the *B. pseudocatenulatum* strains, indicating their direct involvement in the hydrolysis or modification of substrates by these strains. The most abundant GHs gene families in *B. pseudocatenulatum* were GH13, GH43, and GH3, which accounted for 20%, 17%, and 8% of the total GHs genes across the 88 *B. pseudocatenulatum* strains, respectively. Notably, GH2 and GH42 families also appeared in the genomes and accounted for 2% and 3% of the total GHs genes across all of the tested *B. pseudocatenulatum* strains, respectively. As for the twelve *B. pseudocatenulatum* strains used for animal treatment, these different individuals were selected from the four main clusters of *B. pseudocatenulatum* strains and for phylogenetic analysis showed that nearly all the *Bifidobacterium* strains used for analysis in this study were grouped in seven clusters. Further analysis of genomic annotations of 12 *B. pseudocatenulatum* suggested that the genes are rich in MFS transporter, glycosyltransferase and ABC transporter permease (Figure 4c). There were 1286 orthogroups shared across the genomes of the 12 *B. pseudocatenulatum* strains and the distribution of these strain-specific genes in *B. pseudocatenulatum* was diverse, ranging from 37 genes (strain FNXHL2M3) to 208 genes (strain FQHXN83M4) (Figure 4a). Beyond that, a large number of orthogroup unassigned genes (strain-specific genes) of 12 *B. pseudocatenulatum* genomes have been predicted to be involved in glucose metabolism, most of which act through ABC transporters and carbohydrate esterase (Figure 4b). Interestingly, these changes involved in carbohydrate metabolism-related genes are particularly marked in contrast to the rich strain-specific genes in 12 *B. pseudocatenulatum* (Figure 4a,b). Furthermore, among the eight strains of *B. pseudocatenulatum* in the cluster 2 (FHNXY15M2, A14, FFJND17M1, FSHXXA2M9, FAHBZ9L5, FNXHL2M3, FQHXN83M4, and FGSYC12M4), only FAHBZ9L5-specific genes were rich in ABC transporters and carbohydrate esterase, which indicates the potential pathway for polysaccharide utilization by specific genes of *B. pseudocatenulatum* FAHBZ9L5 (Figure 4d). Specifically, carbohydrate transporters on the cell surface capture polysaccharides, and carbohydrate esterase assists in the end digestion of main chain polysaccharides. The primary digested polysaccharides enter the bacteria via ABC transporters, and then are further degraded with the help of GH43 family glycoside hydrolases and α-galactosidase.

A total of 41 antibiotic resistance genes (18 classes) were found across the 88 *B. pseudocatenulatum* strains (Supplementary Material Figure S2). All of the strains shared 17 common resistance genes, including the aminoglycoside resistance genes *gidB*, *rpsL*, and *tlyA*; the fluoroquinolone resistance genes *gyrA*, *gyrB*, and *mfd*; the rifampicin resistance genes *RbpA* and *rpoB*; the antibiotic efflux pump genes *lmrD* and *mtrA*; the lipopeptide resistance gene *rpoC*; the tetracycline resistance gene *rpsJ*; the mupirocin resistance gene *ileS*; the fosfomycin resistance gene *murA*; the trimethoprim resistance gene *dfrE*; the pyrazinamide resistance gene *pncA*; and elongation factor Tu (*EF-Tu*) (Supplementary Material Figure S2).

### 3.4. Effects of B. pseudocatenulatum on the Gut Microbiota in Mice

**Figure 5:**
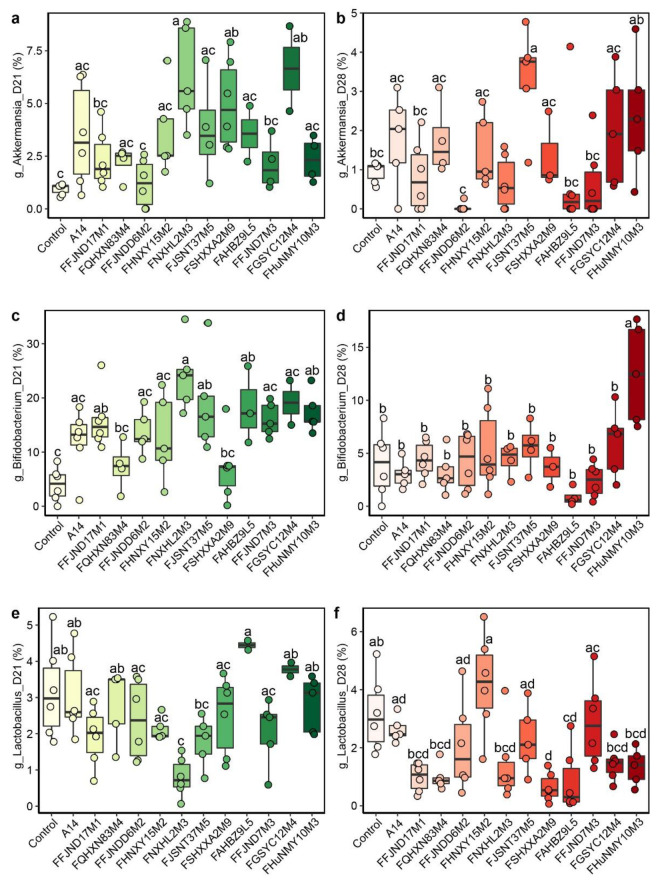
Comparison of different genus in fecal microbiota among groups of mice at different time points (D21, 21 days; D28, 28 days; the relative abundance of genus *Akkermansia* (**a**,**b**), *Bifidobacterium* (**c**,**d**), *Lactobacillus* (**e**,**f**)). Different lowercase letters mean that there is a statistically significant difference between 13 groups at a probability level of *p* < 0.05.

**Figure 6:**
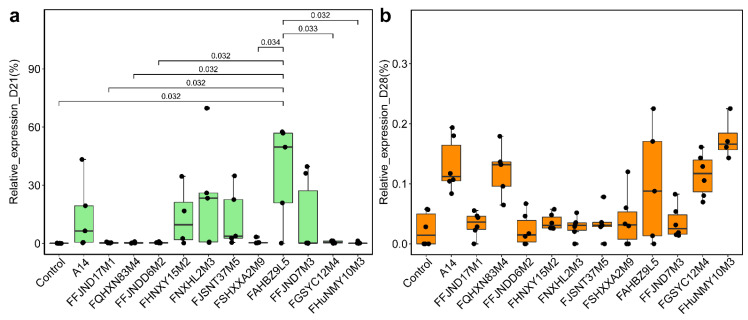
Relative abundance of *B. pseudocatenulatum* species in total *Bifidobacteria* among different mice groups at different time points ((**a**): 21 days; (**b**): 28 days). The *t*-test *p*-values show that this variation was statistically significant (*t*-test *p* < 0.05).

**Figure 7:**
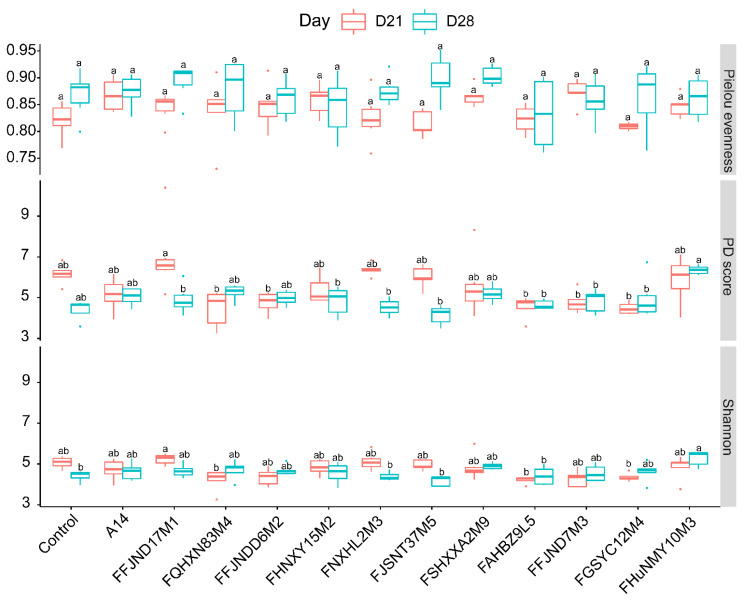
Alpha diversity indexes of gut microbiota in different mice group at different time points (red: 21 days, green: 28 days). Different lowercase letters mean that there is a statistically significant difference between 13 groups at a probability level of *p* < 0.05.

To investigate the effects of 12 *B. pseudocatenulatum* strains from different phylogenetic lineages on the intestinal microecology and carbohydrate metabolism in host, we collected the feces of mice for 16S and *GroEL* amplicon analysis after 3 weeks of intragastric administration and 1 week after cessation of the above-mentioned 12 strain treatments to examine the intestinal colonization ability of the strains. The mean relative abundance of *Bifidobacterium* in the gavage groups was from 0 to 37.03% and significantly different between the groups at 21 days (Figure 5c). The results showed that although most of the *B. pseudocatenulatum* strains had no significant effect on the abundance of *B. pseudocatenulatum* and the α-diversity index of the gut microbiota of healthy mice after 3 and 4 weeks of gavage (Figure S3, Figure 6b and Figure 7), the relative abundance of *B. pseudocatenulatum* in the FAHBZ9L5 group was significantly higher than that in the control, FFJND17M1, FQHXN83M4, FFJNDD6M2, FSHXXA2M9, FGSYC12M4, and FHuNMY10M3 groups at 21 days. After 4 weeks, the relative abundance of *B. pseudocatenulatum* decreased close to 0% in all gavaged groups (Figure 6).

The relative abundance of *Akkermansia* increased in all gavage groups, with notable increases in the FNXHL2M3-, FSHXXA2M9-, and FGSYC12M4-gavaged groups at 21 days. In particular, its relative abundance in the FJSNT37M5-gavaged group was significantly (*p* < 0.05) higher than that in the control group at 28 days, indicating that this strain significantly increased the relative abundance of *Akkermansia* in healthy mice (Figure 5a,b). Most of the groups gavaged with various *B. pseudocatenulatum* strains increased the intestinal flora diversity of *Bifidobacterium* at 21 days. FHUNMY10M3 increased the intestinal flora diversity at both 3 weeks and 4 weeks. FFJND17M1 also initially increased the intestinal flora diversity, but the effect declined after the period of gavage, and the diversity returned to the same level as the control group after 1 week (Figure 5c,d).

**Figure 8:**
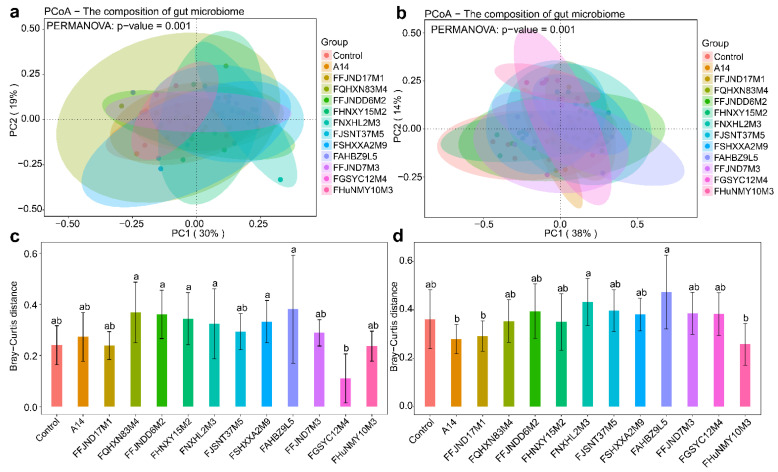
Beta diversity indexes (Bray–Curtis) of gut microbiota in different mice group at different time points ((**a**,**c**): 21 days, (**b**,**d**): 28 days). Different lowercase letters mean that there is a statistically significant difference between 13 groups at a probability level of *p* < 0.05.

The intestinal flora structure of each group was largely similar to that of the control group, with overlap of the PCoA of bacterial community structure plots observed at 21 days (Figure 8a,c). However, the differences in the gut microbiota became larger between most groups at 28 days, particularly between the A14- and FAHBZ9L5-gavaged groups, between the FaHBZ9L5- and FFJND17M1-gavaged groups, and between the FaHBZ9L5- and FHUNMY10M3-gavaged groups (Figure 8b,d).

### 3.5. Effects of B. pseudocatenulatum on Serum Biochemical Indexes and Short Chain Fatty Acids

**Figure 9:**
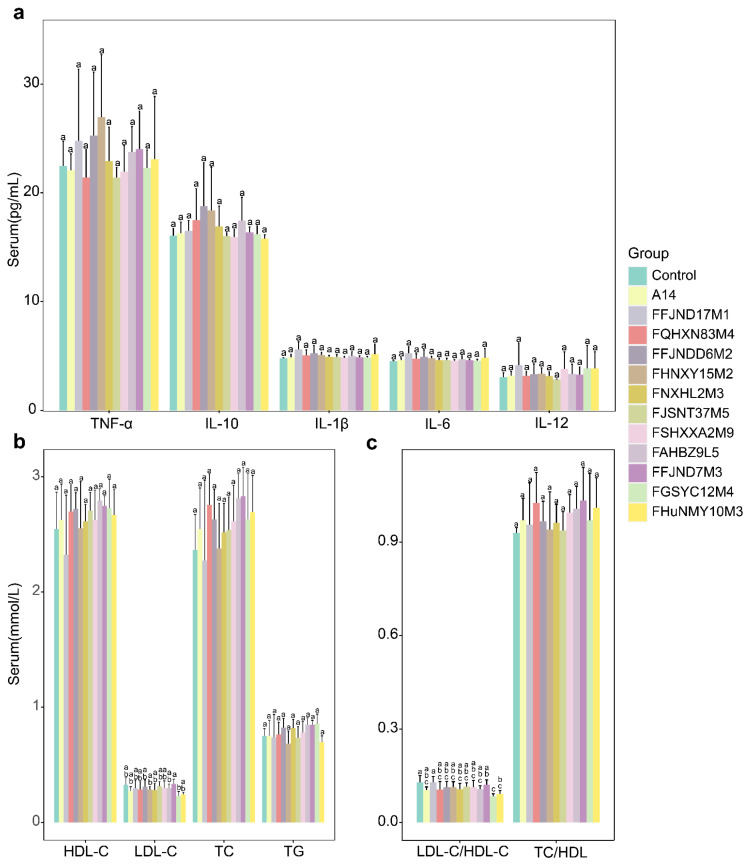
Concentration of serum inflammatory cytokines and serum lipid indexes in different mice groups (**a**–**c**). Different lowercase letters mean that there is a statistically significant difference between 13 groups at a probability level of *p* < 0.05.

**Figure 10:**
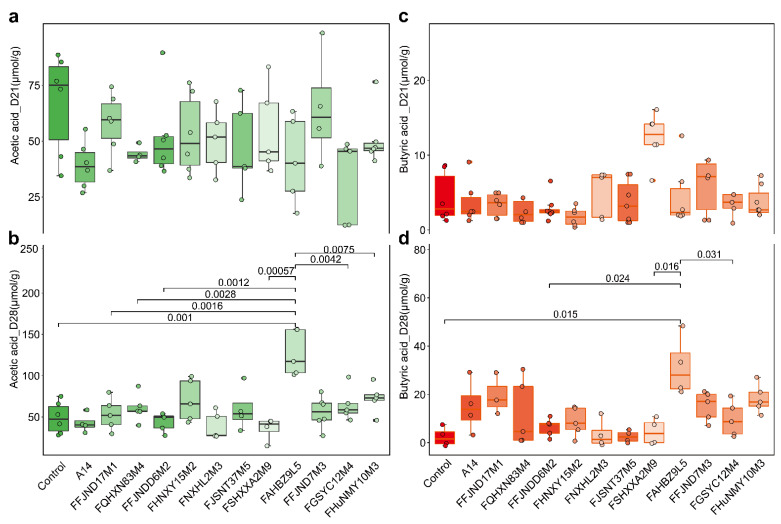
Relative abundance of short chain fatty acids (SCFA) in fecal microbiota among different mice groups at different time points ((**a**,**c**): 21 days; (**b**,**d**): 28 days). Green represents the acetic acid and red representing the butyric acid. The *t*-test *p*-values show that this variation was statistically significant (*t*-test *p* < 0.05).

We determined the serum biochemical indexes after gavage with various *B. pseudocatenulatum* strains. As shown in Figure 9, there is no significant difference in the concentrations of the serum total triglyceride (TG), total cholesterol (TC), high-density cholesterol (HDL-C), low-density lipoprotein cholesterol (LDL-C) levels, tumor necrosis factor-α(TNF-α), interleukin (IL)-10, IL-1β, IL-6, and IL-12 in the strain treated groups, compared to that in the control group after 4 weeks. (*p* < 0.05). At 21 days, the average fecal acetic acid content was 51.18 ± 18.39 μmol/g and butyric acid was 4.43 ± 3.58 μmol/g across all groups, with no significant difference noted among the groups (Figure 10a,c). As shown in Figure S4, there is no significant difference in water and food intake of mice in different 13 groups weekly. (*p* < 0.05).

At 28 days, the fecal acetic acid content was highest in the FAHBZ9L5 group (126.91 ± 27 μmol/g). Specifically, the acetic acid content in the FaHBZ9L5 group increased after the cessation of intragastric administration and was significantly higher than that in other gavaged (FFJND17M1, FQHXN83M4, FFJNDD6M2, FSHXXA2M9, FGSYC12M4 and FHuNMY10M3) and control groups (Figure 10b). At 28 days, the fecal butyric acid content also was highest in the FAHBZ9L5 group (32.33 ± 12.34 μmol/g). Butyric acid was significantly higher in the FAHBZ9L5 group when compared with the control group, FFJNDD6M2, FSHXXA2M9 and FGSYC12M4 group (*p* < 0.05) (Figure 10d).

## 4. Discussion

A comparative genomic analysis for 88 *B. pseudocatenulatum* strains from 397 human and animal fecal samples from different regions in China was performed in this study (Figure S5). Genetic information was obtained using Illumina HiSeq second-generation sequencing, which revealed that the GC content, genome size, and ORF numbers were similar to those of this species previously published in the NCBI database. The sum of all the genes carried by a strain is called the pan-genome, which encompasses the core, dispensable, and unique genomes. The core genome refers to the genes shared by all the individuals of a species, while the unique genome refers to the genes unique to individuals within species [25]. We reconstructed 7977 pan-genes and 1138 core genes from 88 *B. pseudocatenulatum* strains, which showed an open but soon to be closed pan-genome, reflecting their genetic diversity. Core genes are generally involved in important metabolic processes, including amino acid and carbohydrate metabolism, nucleotide transport, translation, and ribosomal biosynthesis. Only 60% of the genes were core genes common to all the strains, and approximately 40% of the genes were specific to individual strains. This suggests that strains can regularly acquire new external genes while adapting to a new environment, which may contribute to the wide distribution of this species in nature.

Phylogenetic trees constructed with direct homologous genes can be used to reliably determine the evolutionary relationships among strains [26]. Using concatenated shared single copy genes, we obtained a well-resolved phylogenetic tree. In this study, strains from northwest China were largely clustered, while those from other regions were scattered among a variety of clusters, which is conjectured to be due to the unique dietary habits that exhibited high consumption of grain food, while consumption of vegetables, fruits, and fish was inadequate in northwest China [27]. However, given the small sample size, substantiating this speculation would require a further survey of dietary habits. The phylogenetic relationships among *B. pseudocatenulatum* strains were independent of host age and sex. Furthermore, the abundance of gene families of carbohydrate-active enzymes had a particular influence on the evolution of these strains. Studies have shown that the extensive use of different carbohydrates by *Bifidobacterium* is a primary reason for its colonization of the human intestinal tract [12,28]. *Bifidobacterium* is widely regarded as an important and broadly distributed intestinal probiotic for carbon metabolism [29]. Significant differences were also present in the number of 10 carbohydrate active enzyme gene families among the *B. pseudocatenulatum* strains derived from the hosts of different age groups, particularly between strains from infants and those from other age groups. The results confirmed that the time from weaning to 3 years of age is a period of rapid changes in the human gut microbiota. Previous studies have mainly focused on the overall changes in the gut microbiota [30], but our study also demonstrated that substantial changes within particular species of symbiotic bacteria also occurred during this period.

Carbohydrate transport and metabolism-related enzymes of *Bifidobacterium pseudocatenulatum* play a very important role in its life activities, and related genes account for the largest proportion. Studies have shown that the difference between species and strains in the carbohydrate utilization capacity of bacteria is related to the presence or absence of specific carbohydrate active enzyme (CAZy) genes [31,32]. Therefore, the carbohydrate-active enzyme genes of 12 strains were further analyzed based on the genome to explore the genes related to the differential response of genes unique to the strains. Carbohydrate esterase (CEs) can remove the ester modification of sugar molecules and participate in side-chain drops to improve the degradation of glycoside hydrolase [33]. Glycosyl transferases (GTs) constitute a large family of enzymes involved in the biosynthesis of oligosaccharides, polysaccharides, and glycoconjugates [34]. Glycosidic hydrolases (GHs) are responsible for the hydrolysis and/or transglycosylation of glycosidic bonds and are mainly involved in the degradation of carbohydrate chains [34]. Recently, three xylosidases from the GH43 family were identified in the genome of a *B. pseudocatenulatu* strain and these enzymes have similar functions in degrading xylan-associated carbon sources [35]. However, the GH43 family mainly encodes arabinoid glycosidase, which facilitates the hydrolysis of oligosaccharides and polysaccharides containing arabinose, thus releasing the available α-arabinose [36]. According to the potential pathway of polysaccharide utilization by specific genes of *B. pseudocatenulatum* FAHBZ9L5 (Figure 4d), the GH43 family and α-galactosidase might favor the strain that hydrolyses carbohydrates containing α-arabinose and α-galactoside, particularly in the gastrointestinal tracts.

The concentrations of SCFA from fermentable carbohydrates in most of the gavaged groups were largely the same as those in the control group, but some strains had lasting effects on the host intestinal tract. The FAHBZ9L5 group significantly increased intestinal acetic acid and butyric acid contents after 4 weeks of treatment. Prior research has shown beneficial effects of acetic acid and butyric acid on host immune regulation and glucose and lipid metabolism [37,38], as well as an ameliorative effect of acetate-producing *B. pseudocatenulatum* on type 2 diabetes [7]. Combining the results of animal experiments and genome analysis, we speculate that the effect of *B. pseudocatenulatum* on the intestinal microecology of healthy hosts was not related to the evolutionary relationships among strains, but rather to the specific genes carried by each strain. The strains with strong colonization ability in the intestines of healthy hosts had more specific genes related to carbohydrate transport and metabolism, which may provide a colonization advantage through substrate competition. FaHBZ9L5 is one of the strains with the greatest number of specific genes among the 12 *B. pseudocatenulatum* (175 specific genes). It showed long-lasting effects on the host’s intestinal microbiota. These effects may be due to this strain’s frequent and prolonged interactions (more than 1 week) with other intestinal microorganisms. The problem of antibiotic resistance gene transfer among intestinal microorganisms has become of great concern in recent years. In this research, regarding *B. pseudocatenulatum* as a potential edible probiotic, most of the assigned resistance genes encode housekeeping bacterial functions (e.g., *gyrA*, *gyrB*, *rpoB* and *rpoC*), so they are not antibiotic resistance genes. Antibiotic resistance is in these cases are associated with particular mutations in these genes rather than their presence (in fact, these are essential genes present in all bacteria). This indicated that most of the resistance genes of this species did not have the potential for horizontal gene transfer (HGT), but this supposition requires further experimental verification.

## 5. Conclusions

The high genetic diversity of *B. pseudocatenulatum* was consistent with its diverse habitats and significant differences were also present in the number of 10 carbohydrate-active enzyme gene families among the strains derived from the hosts of different age groups. This finding supports the inference that through evolution, characteristic enzyme profiles of carbohydrate active gene families have developed in *B. pseudocatenulatum* strains. Comparative genomic analyses suggest that of the 12 screened *B. pseudocatenulatum* strains, specific genes of FAHBZ9L5 are rich in ABC transporters and carbohydrate esterase. Correspondingly, the animal results indicate that FAHBZ9L5 strain supplementation particularly led to a significant increase in acetic acid and butyric acid production. In summary, the preliminary results led us to speculate that strains containing certain gene clusters contribute to carbohydrate utilization in the host, which may play an important role in maintaining competitiveness in the intestine.

## Data Availability

All of the genome sequences used in this study are publicly available in the National Center for Biotechnology Information (NCBI) database; please see PRJNA730686 and supplementary material for detailed information. All supporting data have been provided within the article.

## Supplementary Materials

The following supporting information can be downloaded at: https://www.mdpi.com/article/10.3390/nu14112347/s1, Figure S1: Phylogenetic tree based average nucleotide identity distances between 88 of *B. pseudocatenulatum* strains, Figure S2: Distribution of antibiotic-resistant genes within 88 *B. pseudocatenulatum*, Figure S3: Changes at phylum level of mice fecal microbiota in different groups and at different time points, Figure S4: Water and food intake of mice in different 13 groups. Different lowercase letters mean that there is a statistically significant difference between 13 groups weekly at a probability level of *p* < 0.05, Figure S5: Graphical abstract; Table S1: General genomic features 88 of *B. pseudocatenulatum* strains in this study, Table S2: Animal grouping and treatment, Table S3: General genomic features 13 of *Bifidobacterium* strains in this study.
