# Supplementary material for: The Comparative Analysis of Genomic Diversity and Genes Involved in Carbohydrate Metabolism of Eighty-Eight Bifidobacterium pseudocatenulatum Isolates from Different Niches of China
Source: Nutrients. 2022 Jun 4;14(11):2347. doi: 10.3390/nu14112347 (PMC9183100; doi:10.3390/nu14112347)
Supplement: Supplementary file 1 [file nutrients-14-02347-s001.zip › Supplementary Material.pdf]

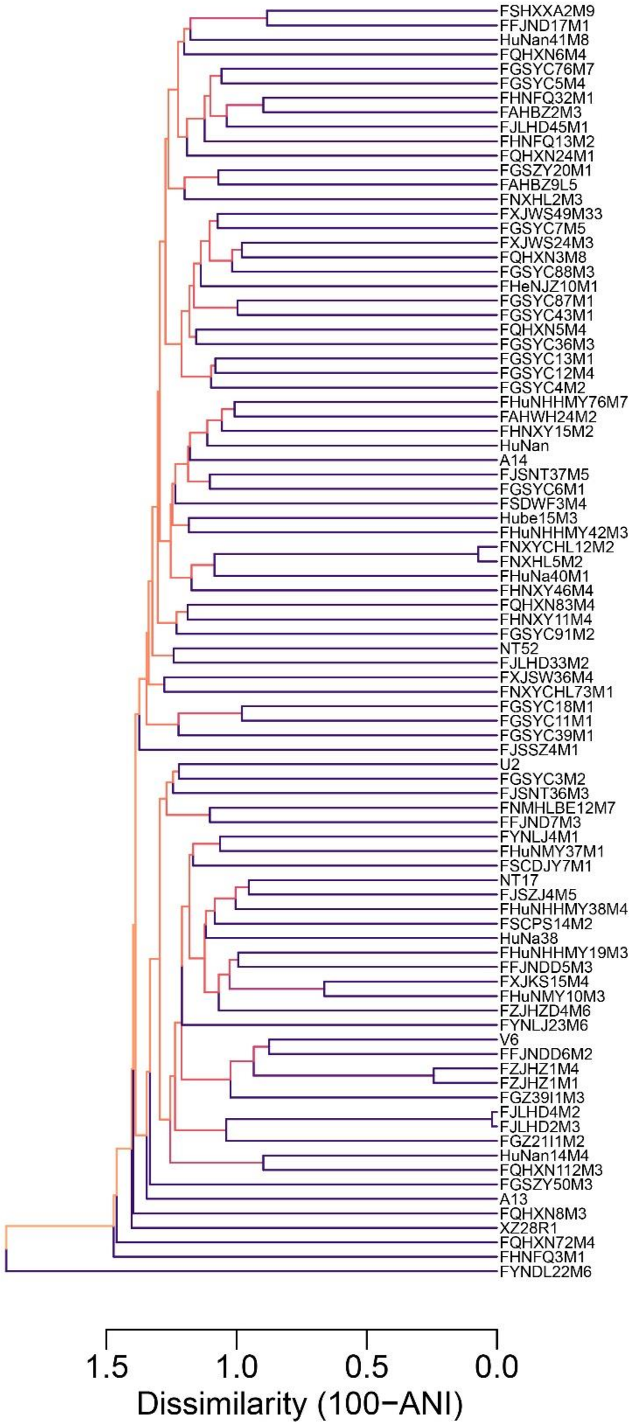

Figure S1. Phylogenetic tree based average nucleotide identity distances between 88 of *B. pseudocatenulatum* strains.

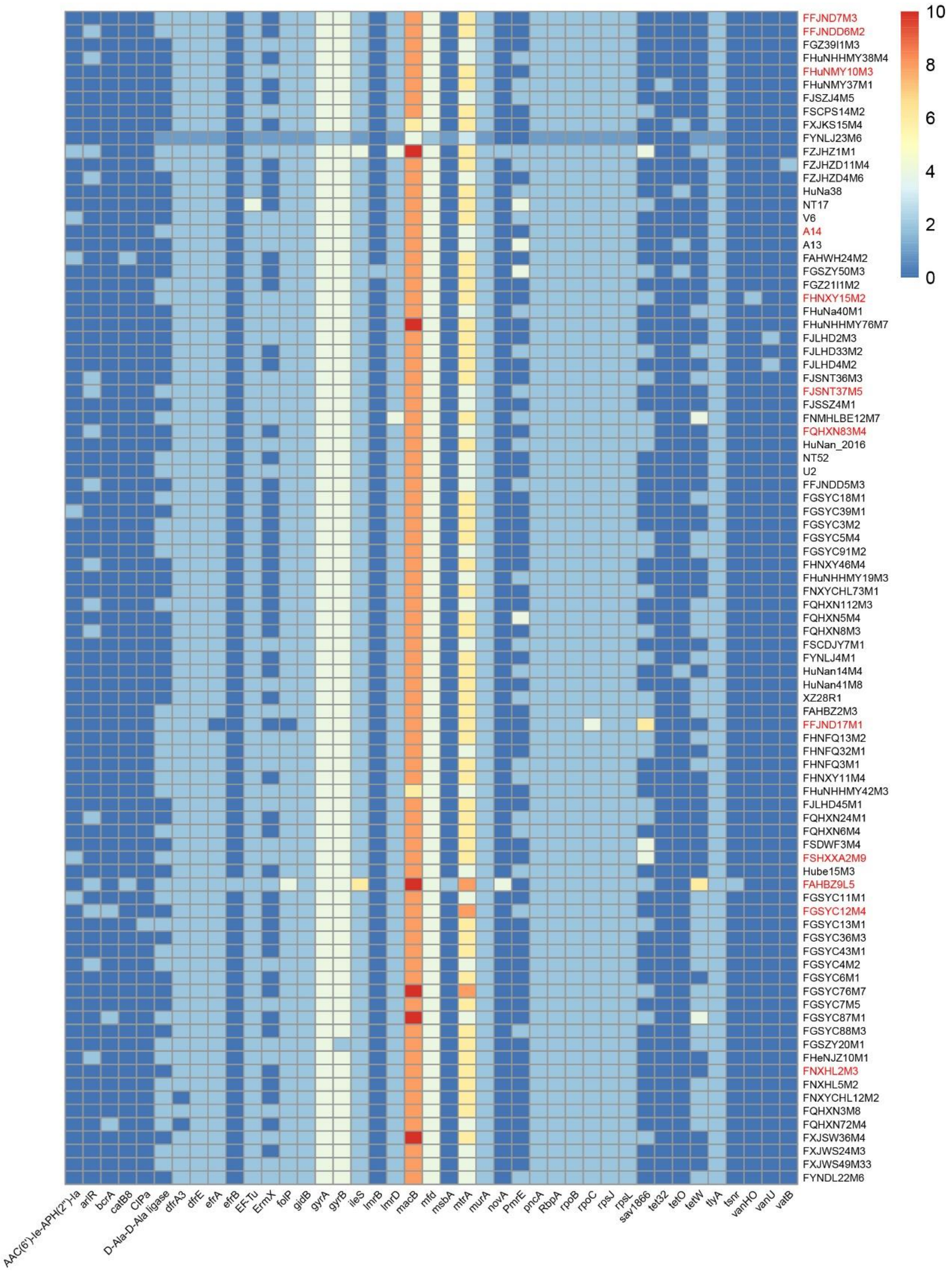

Figure S2. Distribution of antibiotic-resistant genes within 88 *B. pseudocatenulatum*

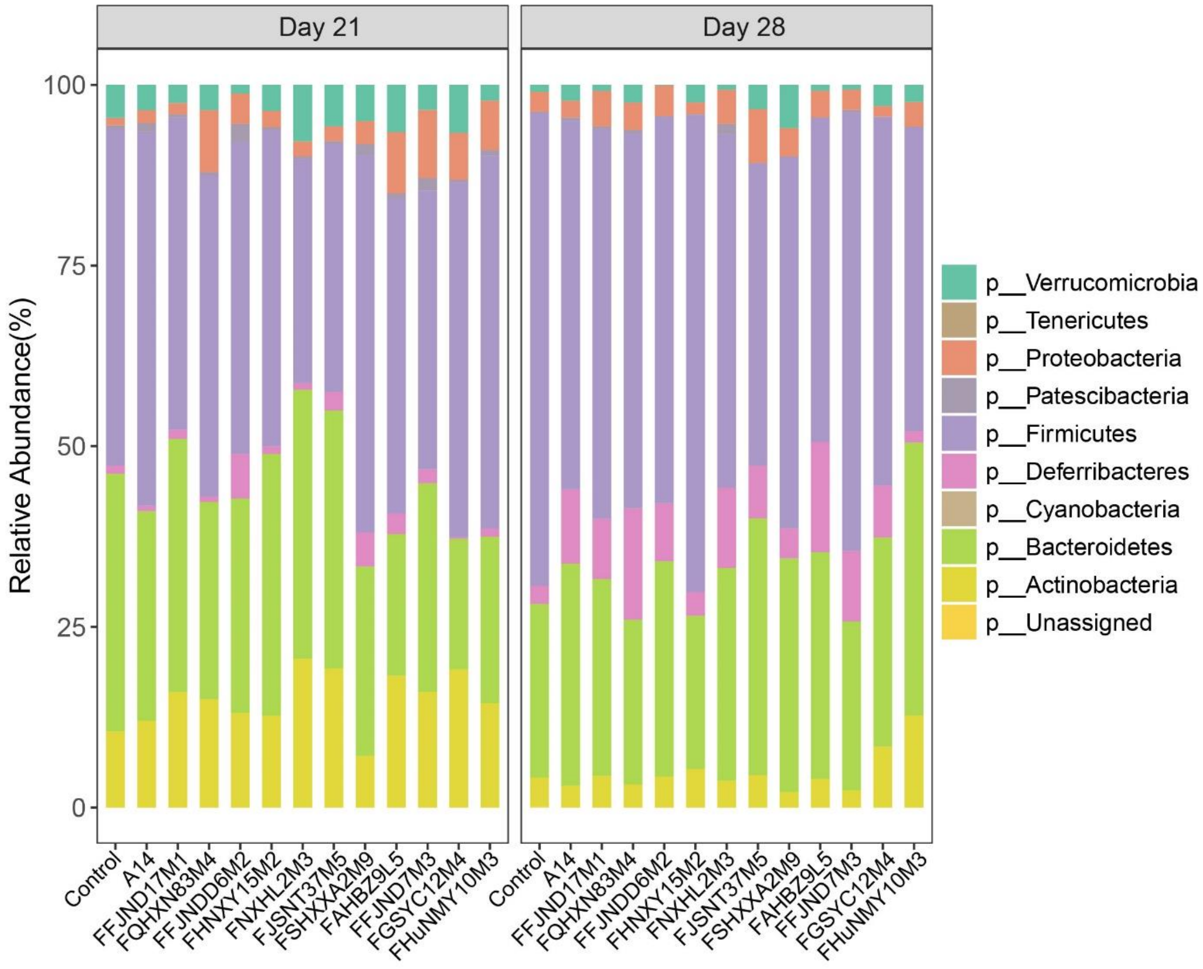

Figure S3. Changes at phylum level of mice fecal microbiota in different groups and at different time points.

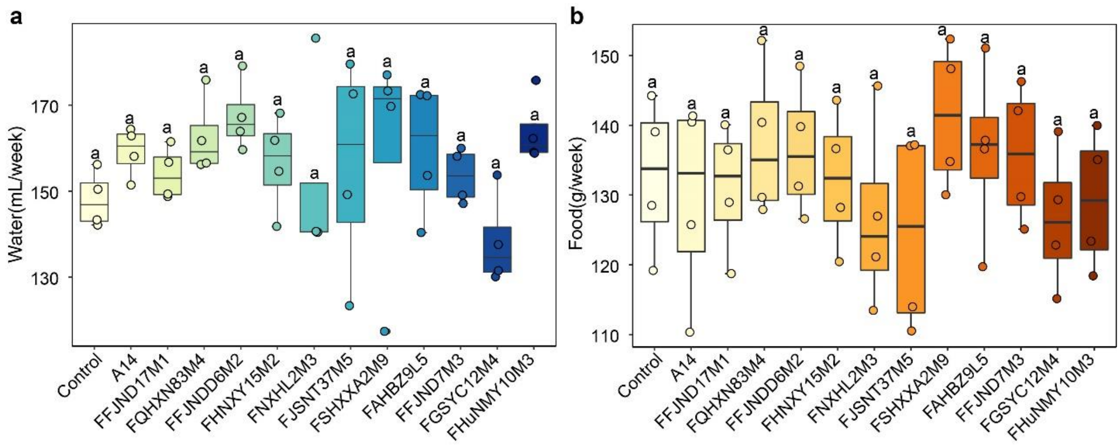

Figure S4. Water and food intake of mice in different 13 groups. Different lowercase letters mean that there is a statistically significant difference between 13 groups weekly at a probability level of $p < 0.05$.

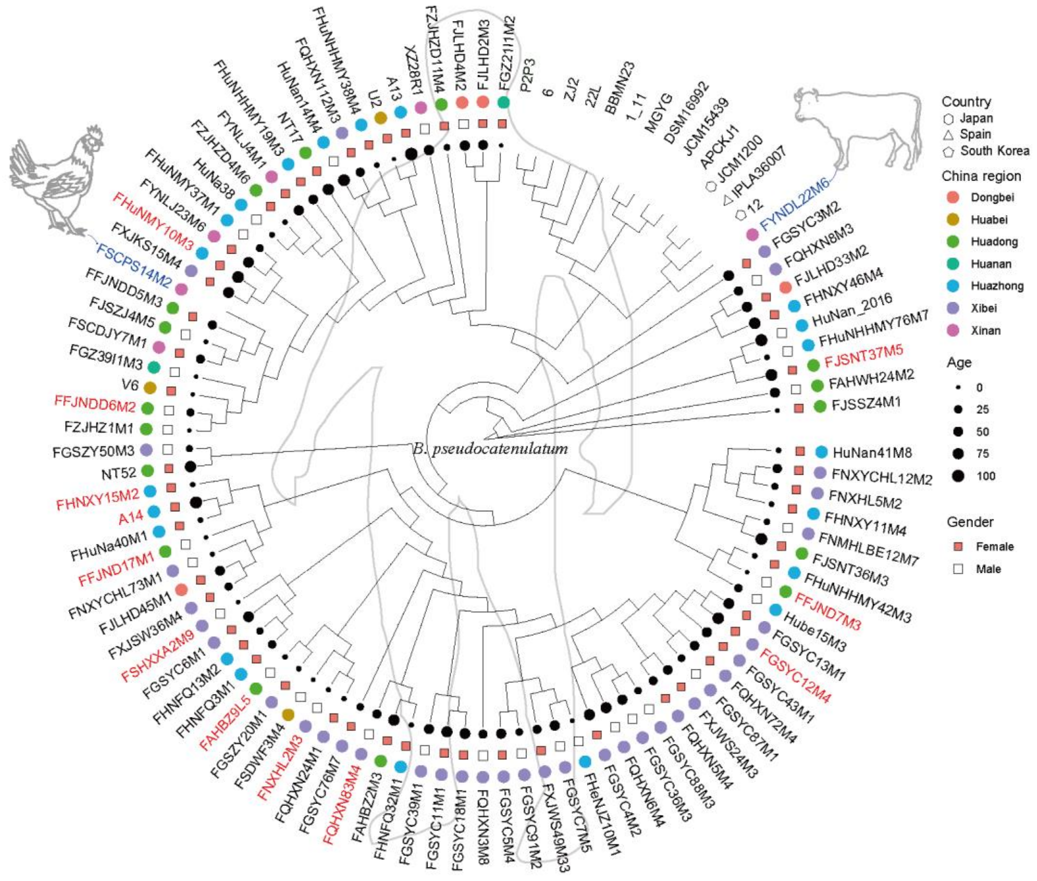

Figure S5. Graphical abstract
