# Supplementary figures and images for: The Comparative Analysis of Genomic Diversity and Genes Involved in Carbohydrate Metabolism of Eighty-Eight Bifidobacterium pseudocatenulatum Isolates from Different Niches of China
Source: Nutrients. 2022 Jun 4;14(11):2347. doi: 10.3390/nu14112347 (PMC9183100; doi:10.3390/nu14112347)

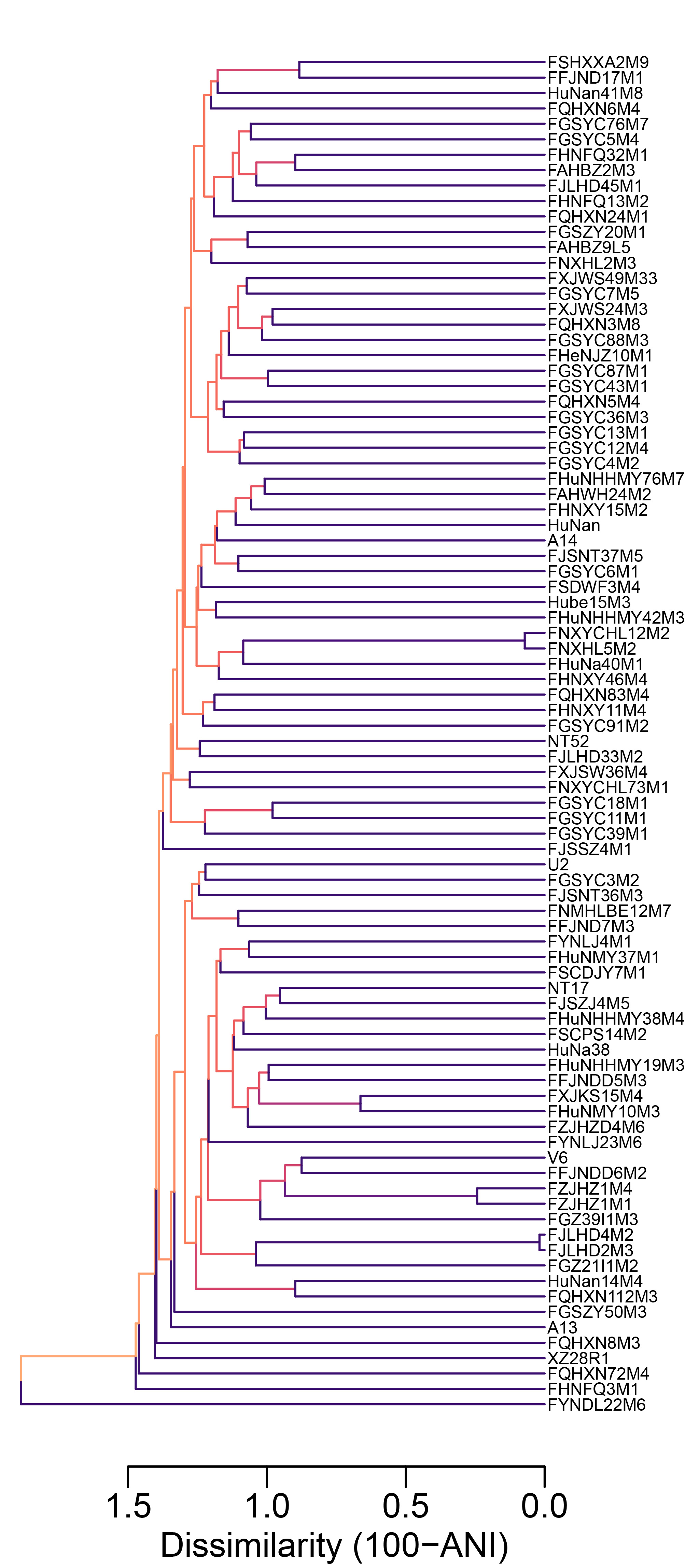

Supplement: Supplementary file 1 [file nutrients-14-02347-s001.zip › Figure S1.jpg]

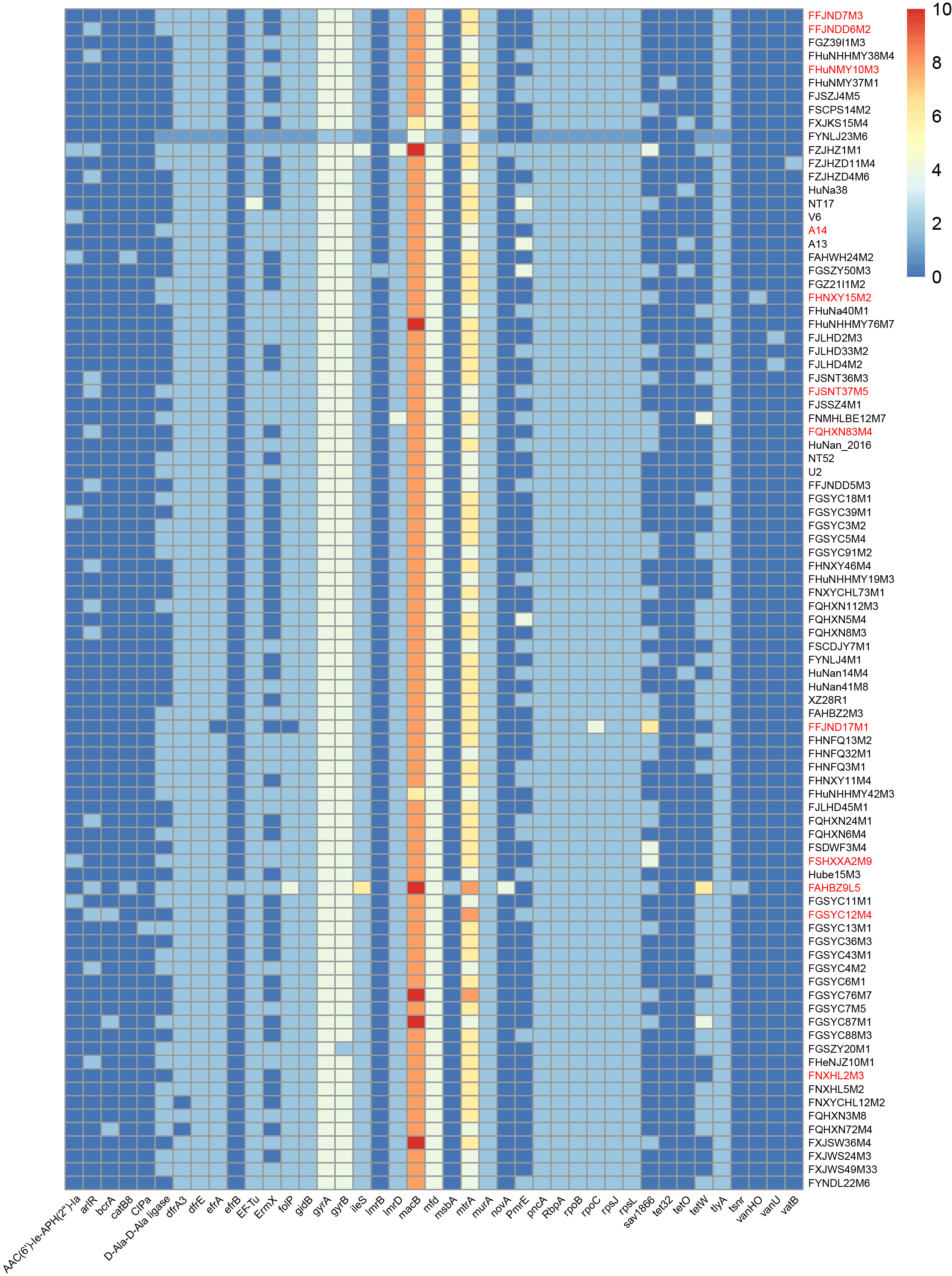

Supplement: Supplementary file 1 [file nutrients-14-02347-s001.zip › Figure S2.jpg]

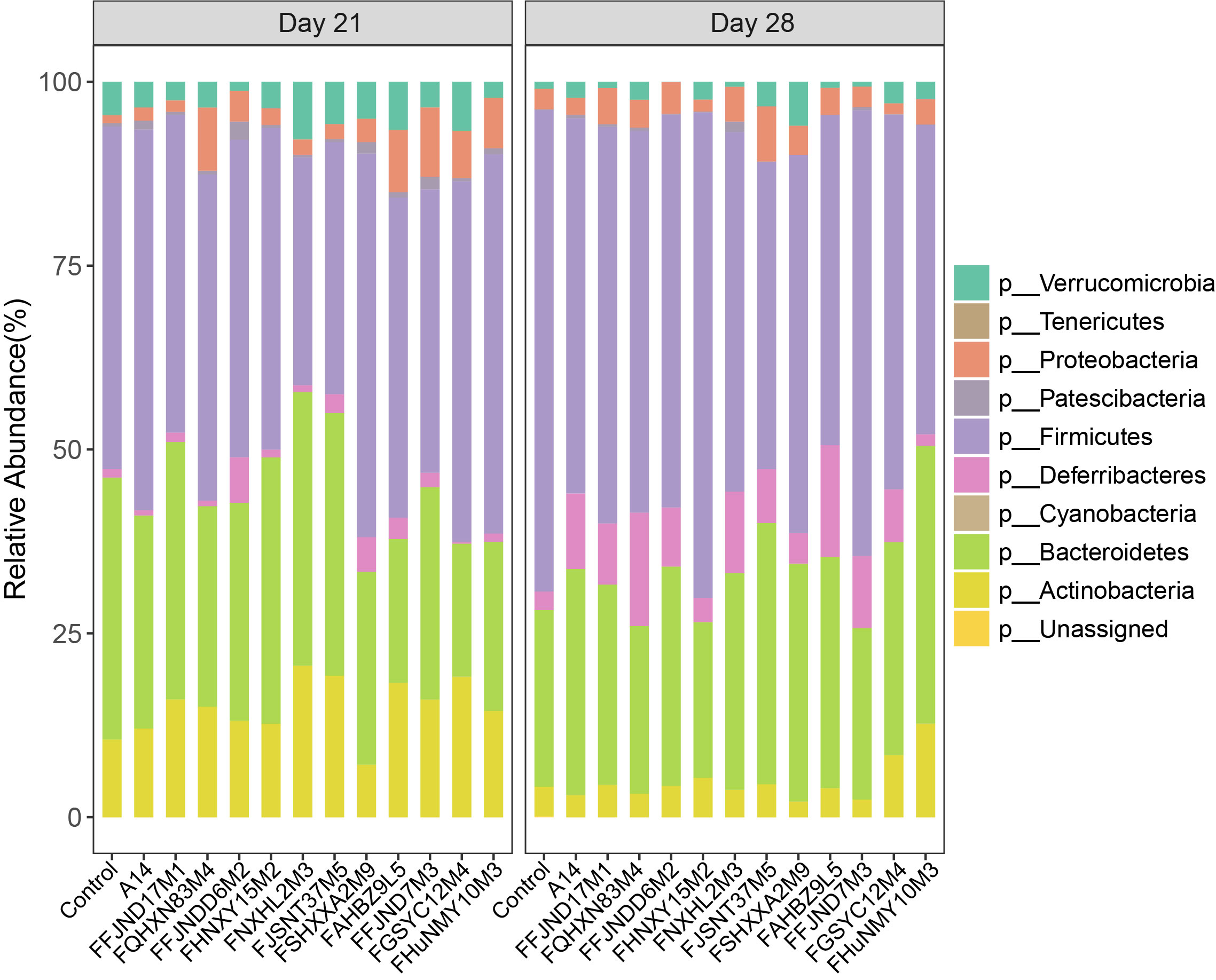

Supplement: Supplementary file 1 [file nutrients-14-02347-s001.zip › Figure S3.jpg]

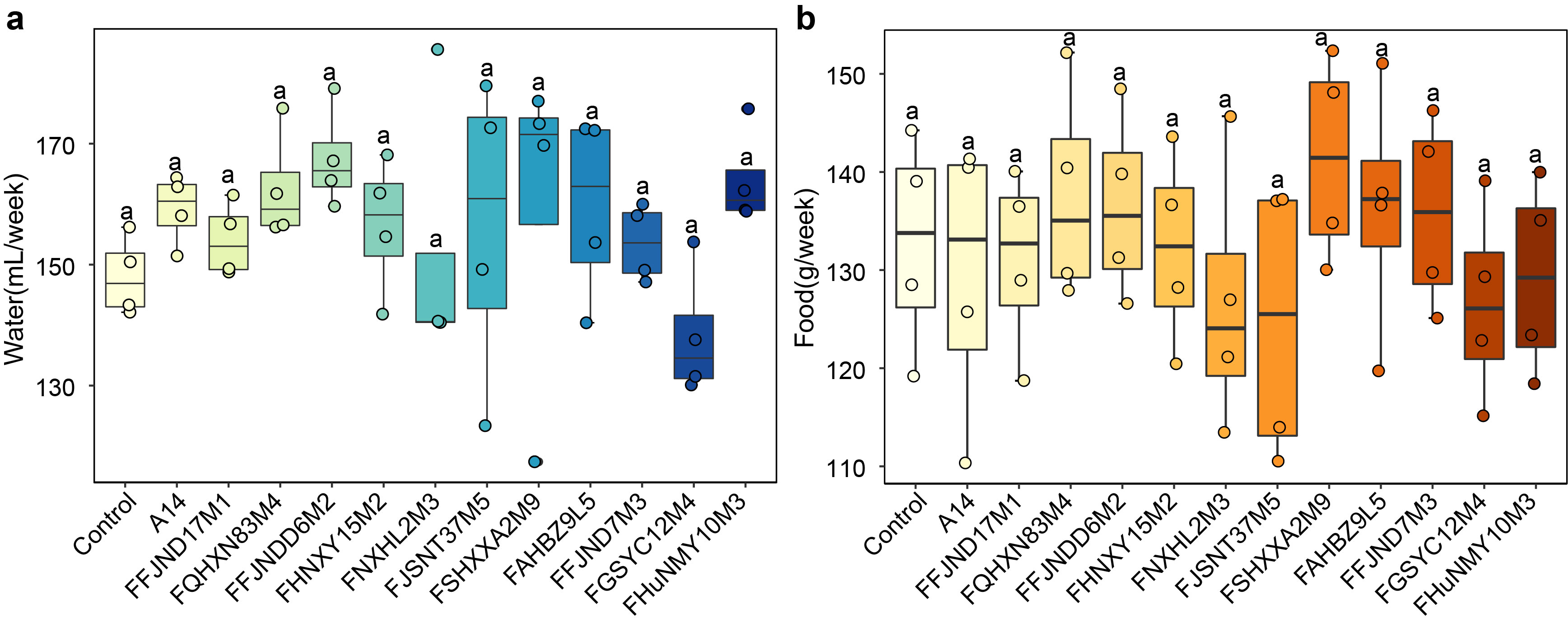

Supplement: Supplementary file 1 [file nutrients-14-02347-s001.zip › Figure S4.jpg]

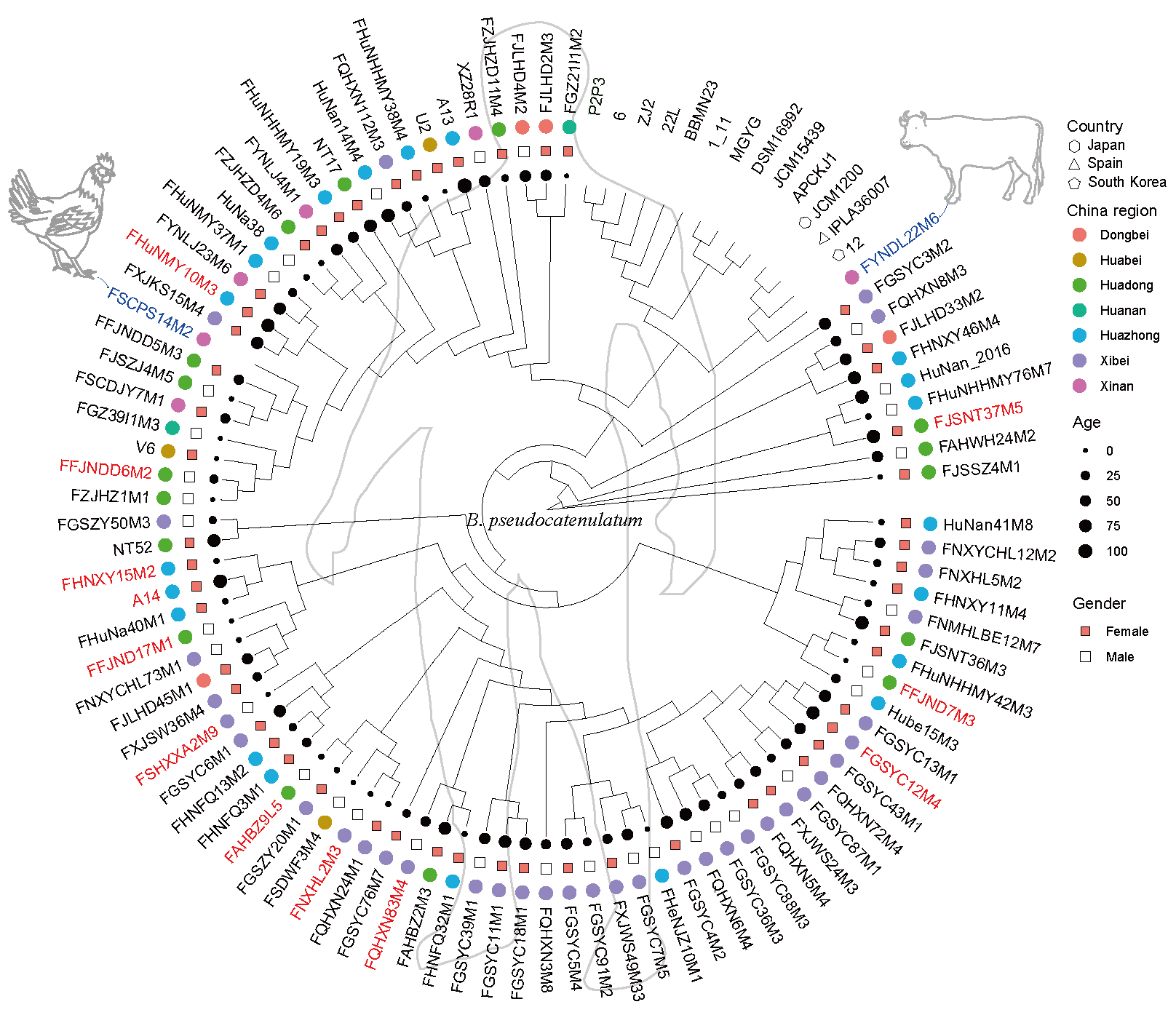

Supplement: Supplementary file 1 [file nutrients-14-02347-s001.zip › Figure S5.jpg]
